# Measurement of Semantic Textual Similarity in Clinical Texts: Comparison of Transformer-Based Models

**DOI:** 10.2196/19735

**Published:** 2020-11-23

**Authors:** Xi Yang, Xing He, Hansi Zhang, Yinghan Ma, Jiang Bian, Yonghui Wu

**Affiliations:** 1 Department of Health Outcomes and Biomedical Informatics University of Florida Gainesville, FL United States

**Keywords:** clinical semantic textual similarity, deep learning, natural language processing, transformers

## Abstract

**Background:**

Semantic textual similarity (STS) is one of the fundamental tasks in natural language processing (NLP). Many shared tasks and corpora for STS have been organized and curated in the general English domain; however, such resources are limited in the biomedical domain. In 2019, the National NLP Clinical Challenges (n2c2) challenge developed a comprehensive clinical STS dataset and organized a community effort to solicit state-of-the-art solutions for clinical STS.

**Objective:**

This study presents our transformer-based clinical STS models developed during this challenge as well as new models we explored after the challenge. This project is part of the 2019 n2c2/Open Health NLP shared task on clinical STS.

**Methods:**

In this study, we explored 3 transformer-based models for clinical STS: Bidirectional Encoder Representations from Transformers (BERT), XLNet, and Robustly optimized BERT approach (RoBERTa). We examined transformer models pretrained using both general English text and clinical text. We also explored using a general English STS dataset as a supplementary corpus in addition to the clinical training set developed in this challenge. Furthermore, we investigated various ensemble methods to combine different transformer models.

**Results:**

Our best submission based on the XLNet model achieved the third-best performance (Pearson correlation of 0.8864) in this challenge. After the challenge, we further explored other transformer models and improved the performance to 0.9065 using a RoBERTa model, which outperformed the best-performing system developed in this challenge (Pearson correlation of 0.9010).

**Conclusions:**

This study demonstrated the efficiency of utilizing transformer-based models to measure semantic similarity for clinical text. Our models can be applied to clinical applications such as clinical text deduplication and summarization.

## Introduction

Semantic textual similarity (STS) is a natural language processing (NLP) task to quantitatively assess the semantic similarity between two text snippets. STS is usually approached as a regression task where a real-value score is used to quantify the similarity between two text snippets. STS is a fundamental NLP task for many text-related applications, including text deduplication, paraphrasing detection, semantic searching, and question answering. In the general English domain, semantic evaluation (SemEval) STS shared tasks have been organized annually from 2012 to 2017 [1-6], and STS benchmark datasets were developed for evaluation [6]. Previous work on STS often used machine learning models [7-9] such as support vector machine [10], random forest [11], convolutional neural networks [12], and recurrent neural networks [13] and topic modeling techniques [8] such as latent semantic analysis [14] and latent Dirichlet allocation [15]. Recently, deep learning models based on transformer architectures such as Bidirectional Encoder Representations from Transformers (BERT) [16], XLNet [17], and Robustly optimized BERT approach (RoBERTa) [18] have demonstrated state-of-the-art performances on the STS benchmark dataset [19] and remarkably outperformed the previous models. More recently, the Text-to-Text Transfer Transformer model [20] and the StructBERT model [21] have further improved the performance on the STS benchmark. These studies demonstrated the efficiency of transformer-based models for STS tasks.

Rapid adoption of electronic health record (EHR) systems has made longitudinal health information of patients available electronically [22,23]. EHRs consist of structured, coded data and clinical narratives. The structured EHR data are typically stored as predefined medical codes (eg, International Classification of Diseases, 9th/10th Revision, codes for diagnoses) in relational databases. Various common data models were used to standardize EHR data to facilitate downstream research and clinical studies [24]. However, clinical narratives are often documented in a free-text format, which contains many types of detailed patient information, such as family history, adverse drug events, and medical imaging result interpretations, that are not well captured in the structured medical codes [25]. As free text, the clinical notes may contain a considerable amount of duplication, error, and incompleteness for various reasons (eg, copy-and-paste or using templates and inconsistent modifications) [26,27]. STS can be applied to assess the quality of the clinical notes and reduce redundancy to support downstream NLP tasks [28]. However, up until now, only a few studies [29-31] have explored STS in the clinical domain due to the limited data resources for developing and benchmarking clinical STS tasks. Recently, a team at the Mayo Clinic developed a clinical STS dataset, MedSTS [32], which consists of more than 1000 annotated sentence pairs extracted from clinical notes. Based on the MedSTS dataset, the 2018 BioCreative/Open Health NLP (OHNLP) challenge [33] was organized as the first shared task examining advanced NLP methods for STS in the clinical domain. In this challenge, two different teams explored various machine learning approaches, including several deep learning models [30,31]. Later, more teams competed in the 2019 National NLP Clinical Challenges (n2c2)/OHNLP STS challenge with a larger clinical STS dataset [34]. During this challenge, many new emerging NLP techniques, such as transformer-based models, were explored.

This study presents our machine learning models developed for the 2019 n2c2/OHNLP STS challenge. We explored state-of-the-art transformer-based models (BERT, XLNet, and RoBERTa) for clinical STS. We systematically examined transformer models pretrained using general English corpora and compared them with clinical transformer models pretrained using clinical corpora. We also proposed a representation fusion method to ensemble the transformer-based models. In this challenge, our clinical STS system based on the XLNet model achieved a Pearson correlation score of 0.8864, ranked as the third-best performance among all participants. After the challenge, we further explored a new transformer-based model, RoBERTa, which improved the performance to 0.9065 and outperformed the best performance (0.9010) reported in this challenge. This study demonstrated the efficiency of transformer-based models for STS in the clinical domain.

## Methods

### Dataset

The 2019 n2c2 organizers developed a corpus of 2054 sentence pairs derived from over 300 million deidentified clinical notes from the Mayo Clinic’s EHR data warehouse. The sentence pairs were divided into a training set of 1642 sentence pairs for model development and a test set of 412 sentence pairs for evaluation. Similar to the annotation scheme in the general English domain, the challenge corpus was annotated by assigning a similarity score for each sentence pair as a number on a scale from 0.0 to 5.0, where 0.0 indicates that the semantics of the two sentences are entirely independent (ie, no overlap in their meanings), and 5.0 signifies that two sentences are semantically equivalent. Annotators used arbitrary similarity scores between 0.0 and 5.0, such as 2.5 or 3.5, to reflect different levels of equality. Table 1 presents the descriptive statistics of the datasets. The distribution of similarity scores is quite different between the training and test datasets. In the training set, the range with the most cases (509/1642, 31.0%) was (3.0, 4.0], whereas in the test set, most scores (238/412, 57.8%) were distributed in the range (0.0, 1.0]. In this study, we denoted this challenge dataset as STS-Clinic. In addition to the STS-Clinic, we also used a general English domain STS benchmark dataset from the SemEval 2017 [6] as an external source. We merged the original training and development datasets to create a unique dataset of 7249 annotated sentence pairs. We denoted this combined general English domain dataset as STS-General and used it as a complementary training set for model development in this study. Compared to the STS-Clinic, the similarity scores in STS-General were more evenly distributed in different ranges (Table 1).

**Table 1:** Descriptive statistics of the datasets.

Dataset	Sentence pairs, n	Annotation distribution, n (%)				
		[0.0, 1.0]	(1.0, 2.0]	(2.0, 3.0]	(3.0, 4.0]	(4.0, 5.0]
STS-Clinic^a^ Training	1642	312 (19.0)	154 (9.4)	394 (24.0)	509 (31.0)	273 (16.6)
STS-Clinic Test	412	238 (57.8)	46 (11.2)	32 (7.8)	62 (15.0)	34 (8.3)
STS-General Training	7249	1492 (20.6)	1122 (15.5)	1413 (19.5)	1260 (17.4)	1962 (27.1)

^a^STS: semantic textual similarity.

### Preprocessing of Sentence Pairs

We developed a preprocessing pipeline to normalize each sentence pair, including (1) converting all words to lower case; (2) inserting white spaces to separate words from punctuation (eg, “[ab/cd]” → “[ ab / cd ]”; “abc,def” → “abc , def”); and (3) replacing two or more spaces or tabs (“\t”) with a single space. We did not remove any stop-words from the sentences and kept the original formats of the numbers without any conversion. Since different transformer models adopted different tokenization strategies (eg, WordPiece for BERT, byte pair encoding for RoBERTa, and SentencePiece for XLNet), our preprocessing automatically picked the appropriate tokenizer according to the transformer model in use.

#### Transformer Model-Based STS System

In this study, we investigated three transformer models (BERT, XLNet, and RoBERTa) for clinical STS. BERT is a bidirectional transformer-based encoder model pretrained with a combination of masked language modeling (MLM) and next sentence prediction. RoBERTa has the same architecture as BERT but pretrained with a robust optimizing strategy. The RoBERTa pretraining procedure used dynamic MLM but removed the next sentence prediction task. XLNet is a transformer-based model pretrained with the bidirectional autoregressive language modeling method. Unlike the MLM used by BERT and RoBERTa, the autoregressive language model uses data permutation instead of data corruption and reconstruction. All three transformer models provided two different settings: a “BASE” setting and a “LARGE” setting. The main difference between the BASE model and the LARGE model is the number of layers. For example, the BERT-base model features 12 layers of transformer encoder layers, 768 hidden units in each layer, and 12 attention heads, while the BERT-large consists of 24 transformer blocks with a hidden size of 1024 and 16 attention heads. The total number of parameters for the BERT-large model is approximately 340 million, which is about 3 times more than the BERT-base model. In this study, we explored general transformers (pretrained using general English corpora) using both the BASE model and the LARGE model. We also examined clinical transformers pretrained using clinical notes from the MIMIC-III database. For clinical transformers, we adopted the BASE settings as we did not observe additional benefits from using the LARGE setting.

**Figure 1:**
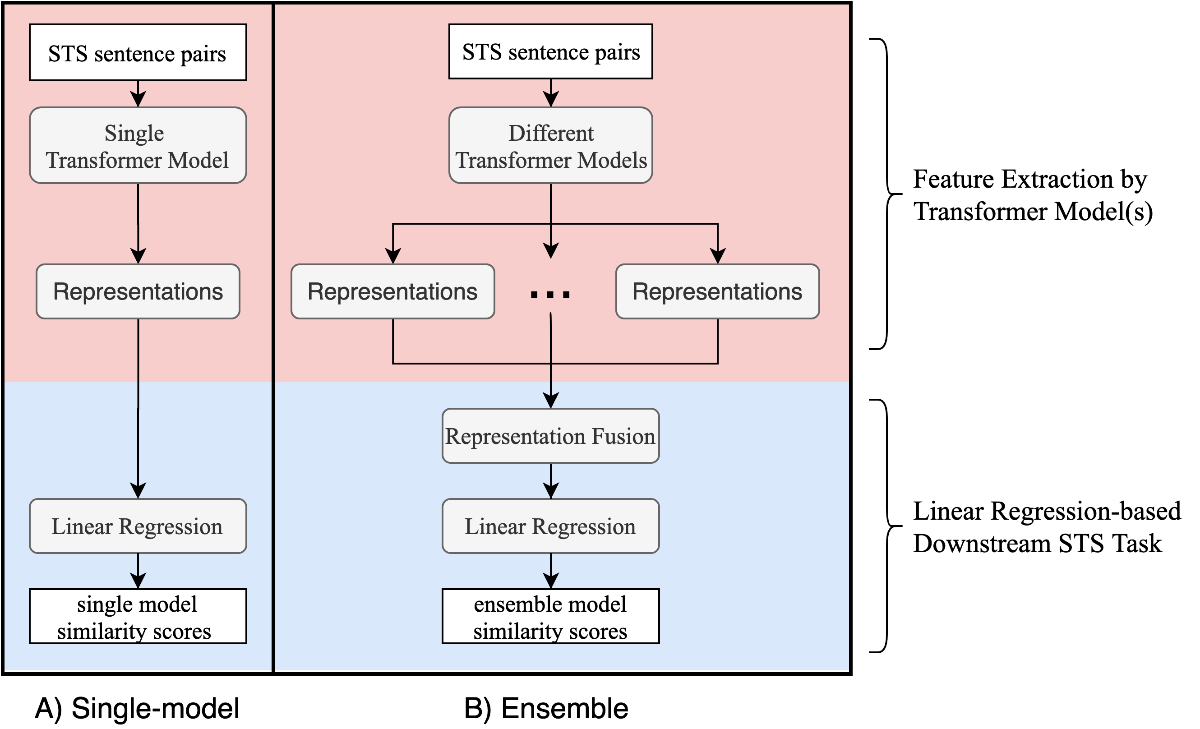
An overview of our single-model and ensemble solutions for clinical STS. STS: semantic textual similarity.

As shown in Figure 1, our STS system has two modules: (1) a transformer model–based feature learning module and (2) a regression-based similarity score learning module. In the feature learning module, transformer-based models were applied to learn distributed sentence-level representations from sentence pairs. In the similarity score learning module, we adopted a linear regression layer to calculate a similarity score between 0.0 and 5.0 according to the distributed representations derived from the transformers. We explored both single-model and ensemble solutions. Figure 1A shows the single-model solution where only one transformer-based model was used for feature representation learning. Figure 1B shows the ensemble solution where different transformer models were integrated. Ensemble learning is an efficient approach to aggregate different machine learning models to achieve better performance [35]. In this work, we tried different strategies to combine the distributed representations from two or three transformers as a new input layer for the similarity score learning module. We explored several methods to combine the distributed representations from different transformers, including (1) simple head-to-tail concatenation, (2) pooling, and (3) convolution.

#### Training Strategy

**Figure 2:**
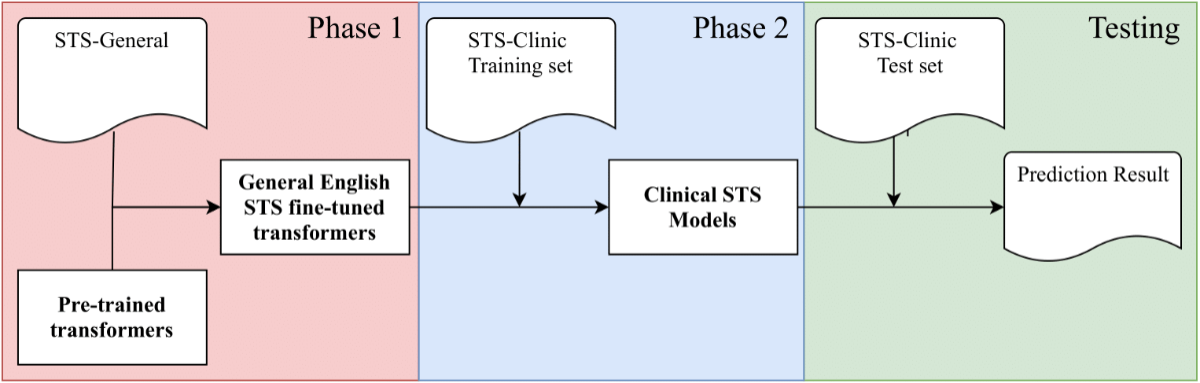
The two-stage procedure for clinical STS model development. STS: semantic textual similarity.

As shown in Figure 2, we adopted a two-phase procedure to train our clinical STS models. In the first phase, an intermediate STS model was fine-tuned using the STS-General corpus. Subsequently, the intermediate model was further fine-tuned using the STS-Clinic corpus in phase 2. The fine-tuned model from the second phase was used for final testing. We used 5-fold cross-validation for hyperparameter optimization in both phase 1 and phase 2 training. We optimized the epoch number, batch size, and learning rate according to the cross-validation results.

#### Experiments and Evaluations

In this study, we implemented our STS system using the Transformers library developed by the HuggingFace team [36]. We also used the PyTorch-based general transformer models trained using general English corpora maintained by the HuggingFace team. The clinical transformer models were derived by further pretraining these general transformer models with clinical notes from the MIMIC-III database [37]. Table 2 shows the hyperparameters used for each transformer model. For evaluation, the results were calculated as the Pearson correlation scores using the official evaluation script provided by the 2019 n2c2/OHNLP challenge organizers. To report the *P* value for each Pearson correlation score, we adopted the SciPy package [38].

**Table 2:** Hyperparameters for transformer models.

Model	Number of epochs	Batch size	Learning rate^a^
BERT-base^b^	4	8	1.00E-05
BERT-mimic	3	8	1.00E-05
BERT-large	3	8	1.00E-05
XLNet-base	3	4	1.00E-05
XLNet-mimic	3	4	1.00E-05
XLNet-large	4	4	1.00E-05
RoBERTa-base^c^	3	4	1.00E-05
RoBERTa-mimic	3	4	1.00E-05
RoBERTa-large	3	4	1.00E-05
BERT-large + XLNet-large	4	8	1.00E-05
BERT-large + RoBERTa-large	3	4	1.00E-05
RoBERTa-large + XLNet-large	4	4	1.00E-05
BERT-large + XLNet-large + RoBERTa-large	3	2	1.00E-05

^a^The learning rate is a tuning parameter in an optimization algorithm that determines the step size at each iteration while moving toward a minimum of a loss function [39].

^b^BERT: Bidirectional Encoder Representations from Transformers.

^c^RoBERTa: Robustly optimized BERT approach.

## Results

Table 3 compares the performance of the different transformer models on the test dataset. The RoBERTa-large model achieved the best Pearson correlation of 0.9065 among all models, which outperformed the two models we developed and submitted during the challenge, including the XLNet-large (a Pearson correlation score of 0.8864) and the BERT-large models (a Pearson correlation score of 0.8549). For RoBERTa and XLNet, the models developed using the LARGE setting pretrained using general English corpora achieved better performances than their BASE settings (0.9065 vs 0.8778 for RoBERTa; 0.8864 vs 0.8470 for XLNet, respectively), whereas the BERT-base achieved a Pearson correlation score of 0.8615 that outperformed the BERT-large model’s score of 0.8549. For all transformers, the models pretrained using general English corpora (in both LARGE settings and BASE settings) outperformed their corresponding clinical models pretrained using clinical notes from the MIMIC-III database. Among the ensemble models, the BERT-large + RoBERTa-large model achieved the best Pearson correlation score of 0.8914, which is remarkably lower than the best model, RoBERTa-large. We also observed that the performances of ensemble models were often in between the two individual models (eg, BERT-large + RoBERTa-large achieved 0.8914, which is between the BERT-large score of 0.8549 and RoBERTa-large score of 0.9065). The ensemble model of all three transformers achieved a Pearson correlation of 0.8452, which was even worse.

**Table 3:** Performances of the Pearson correlation on the test set.

Model	Pearson correlation on test set	*P* value
BERT-base^a^	0.8615	<.001
BERT-mimic	0.8521	<.001
BERT-large^b^	0.8549	<.001
XLNet-base	0.8470	<.001
XLNet-mimic	0.8286	<.001
XLNet-large^b,c^	0.8864	<.001
RoBERTa-base^d^	0.8778	<.001
RoBERTa-mimic	0.8705	<.001
RoBERTa-large	*0.9065*	<.001
BERT-large + XLNet-large^b^	0.8764	<.001
BERT-large + RoBERTa-large	0.8914	<.001
RoBERTa-large + XLNet-large	0.8854	<.001
BERT-large + XLNet-large + RoBERTa-large	0.8452	<.001

^a^BERT: Bidirectional Encoder Representations from Transformers.

^b^The challenge submissions.

^c^The best challenge submission (ranked 3rd).

^d^RoBERTa: Robustly optimized BERT approach.

## Discussion

### Principal Results

Clinical STS is a fundamental task in biomedical NLP. The 2019 n2c2/OHNLP shared task was organized to solicit state-of-the-art STS algorithms in the clinical domain. We participated in this challenge and developed a deep learning–based system using transformer-based models. Our best submission (XLNet-large) achieved the third-best performance (a Pearson correlation score of 0.8864) among the 33 teams. Based on our participation, we further explored RoBERTa models and improved the performance to 0.9065 (RoBERTa-large), demonstrating the efficiency of transformer models for clinical STS. We also further explored three different ensemble strategies to develop ensembled models using transformers. Our experimental results show that the ensemble methods did not outperform the unified individual models. Another interesting finding is that the transformers pretrained using the clinical notes from the MIMIC-III database did not outperform the general transformers pretrained using general English corpora on clinical STS. One possible reason might be that the clinical corpora we used for training are relatively small compared with the general English corpus. Further investigation examining these findings is warranted.

### Experiment Findings

Although previous studies [40-44] have shown that pretraining transformer models with domain-specific corpora could enhance their performances in domain-related downstream tasks (such as clinical concept extraction), our results in this study indicated that this strategy might not be helpful for clinical STS. For all three types of transformers explored in this study, the models pretrained using general English text consistently obtained higher scores than the corresponding models pretrained using clinical text. For example, the Pearson correlation score achieved by the RoBERTa-mimic was 0.8705; however, the RoBERTa-base yielded a higher performance of 0.8778. Tawfik et al [45] have similarly observed that the PubMed pretrained BioBERT did not outperform the corresponding general BERT model pretrained using English text on clinical STS.

In the clinical STS task, using STS-General (an STS corpus annotated in the general English domain) as an extra training set in addition to STS-Clinic could efficiently improve performances for transformer-based models. Taking the RoBERTa model as an example, the RoBERTa-large fine-tuned using only the clinical text (ie, STS-Clinic) achieved a Pearson correlation score of 0.8720; however, the same model fine-tuned with both the general English text (ie, STS-General) and clinical text (ie, STS-Clinic) achieved a score of 0.9065 (approximately 0.035 higher). We observed similar results for BERT and XLNet. Without Phase 1 (Figure 2), the BERT-large and XLNet-large models achieved Pearson correlation scores of 0.8413 and 0.8626, respectively, which are lower than the results we submitted (0.8549 and 0.8864) using two-phase training. We looked into the training datasets for possible reasons. Although the STS-General and STS-Clinic were extracted from different domains, there are common contents shared between them. First, the annotation guidelines between the two datasets were highly aligned. For both datasets, the annotation scale is from 0.0 to 5.0, and each score reflects the same similarity level. Since the two STS datasets were annotated by different annotators, subjective annotation bias might be introduced (eg, the judgement and agreement of semantic similarity among annotators might be different in the two datasets). However, our experiment results showed that training with both datasets improved the performance despite the potential annotation bias. Second, a considerable portion of STS-Clinic sentence pairs are common descriptions that do not require comprehensive clinical knowledge to interpret the semantics. Typical examples include sentences extracted from Consultation Note or Discharge Summary as follows:

Plan: the patient stated an understanding of the program, and agrees to continue independently with a home management program.

Thank you for choosing the name M.D. care team for your health care needs!

On the other hand, there are many sentences in the STS-General associated with healthcare. An example is exhibited below:

Although obesity can increase the risk of health problems, skeptics argue, so do smoking and high cholesterol.

Tang et al [30] have demonstrated that combining representations derived from different models is an efficient strategy in clinical STS. We explored similar strategies to combine sentence-level distributed representations, including vector concatenation, average pooling, max pooling, and convolution. Surprisingly, our results showed that such ensemble strategies did not help transformer-based STS systems. For example, for the ensemble model derived from the BERT-large and the XLNet-large models (ie, BERT-large + XLNet-large), the achieved Pearson correlation scores for vector concatenation, average pooling, max pooling, and convolution were 0.8764, 0.8760, 0.8799, and 0.8803, respectively. All the results were approximately 0.01 lower than that for XLNet-large (0.8864). We also observed that ensemble models’ performances were consistently in between the two individual models (0.8549 for BERT-large and 0.8864 for XLNet-large). Future studies should examine this finding.

To examine the statistical significance among different models’ results, we used a 1-tailed parametric test based on the Fisher Z-transformation [46], adopted in the previous SemEval STS shared tasks [2-4]. Our best model (ie, RoBERTa-large) achieved a statistically significant higher performance than most of our other solutions (see Multimedia Appendix 1) but was not significantly better than the models XLNet-large (*P*=.07), BERT-large + RoBERTa-large (*P*=.13), and RoBERTa-large + XLNet-large (*P*=.06). The significance analysis indicated that these four models performed very similarly to each other.

### Error Analysis

We compared the system prediction from our best model (ie, RoBERTa-large) with the gold standards and identified sentence pairs with the largest discrepancy in terms of the similarity score. Among the top 50 sentence pairs, 26 of them had labeled scores in the range of 0.0 to 1.0, and only 6 sentence pairs had gold standard STS scores over 3.0. We further split the testing results into two subsets using a threshold score of 2.5 on gold standards and calculated the mean and median of the differences between the gold standards and predictions. For the subgroup consisting of sentence pairs with gold standard scores over 2.5, the mean and median of difference were 0.46 and 0.37. For the other subset (difference≤2.5), the mean and median of difference were 0.69 and 0.66. Therefore, it was more challenging for the system to predict appropriate STS scores for sentence pairs with low similarity (gold standard score≤2.5) than for those with high similarity.

**Figure 3:**
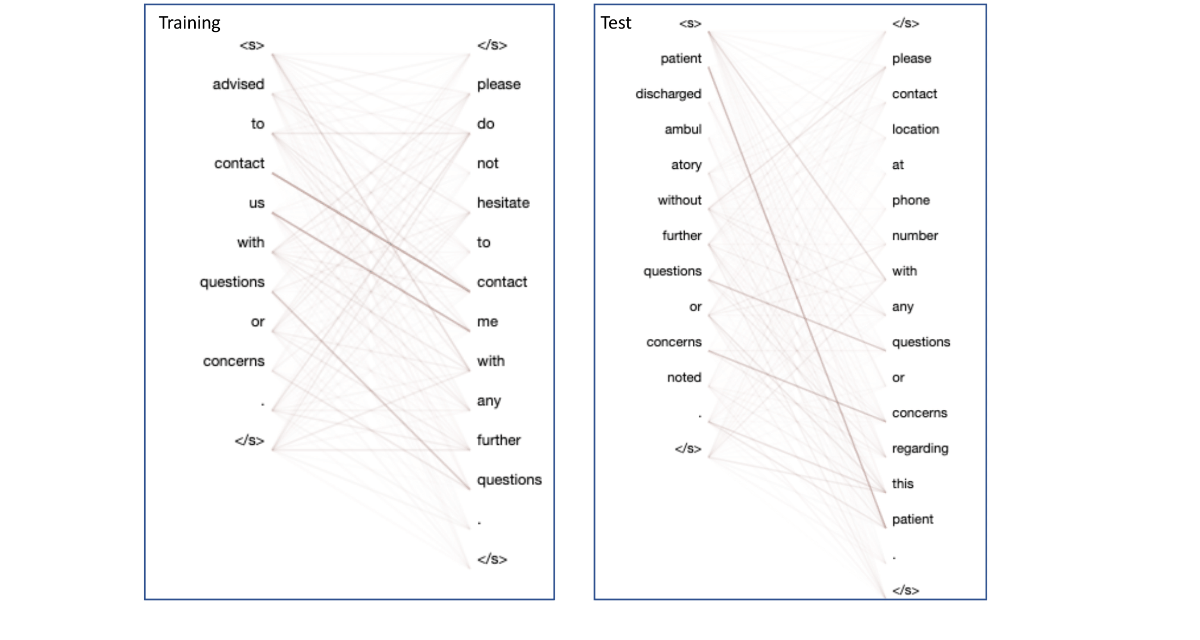
Transformer model attention visualization on two examples from STS-Clinic. STS: semantic textual similarity.

We also observed that sentence pairs with high similarity scores usually have a similar sentence structure where many words occur in both sentences. Therefore, we hypothesized that the STS models will assign higher scores to sentence pairs that share a large portion of their lexicons and similar syntax. To test our hypothesis, we adopted the BertViz package [47] to profile the attention pattern of the RoBERTa-large model (ie, our best STS model). BertViz can generate the attention pattern between two sentences by linking words via lines, where the line weights reflect the attention weights; higher line weights indicate higher attention weights between the two words. Table 4 and Figure 3 show an example for two sentence pairs on a similar topic from the training and test sets. In the first example from the training set, the attention pattern has three dominant attention weights (eg, “questions-questions”) and the similarity score for this sentence pair is labeled as 5.0. However, the attention pattern for the sentence pair from the test set also has similar dominant attention weights (such as “questions-questions”) but was labeled with a similarity score of 0.0.

**Table 4:** Transformer model attention visualization on two examples from STS-Clinic.

Category	Sentence pair	Gold standard	Prediction
Training	S1^a^: advised to contact us with questions or concerns. S2: please do not hesitate to contact me with any further questions.	5	N/A^b^
Test	S1: patient discharged ambulatory without further questions or concerns noted. S2: please contact location at phone number with any questions or concerns regarding this patient.	0	2.5

^a^S: sentence.

^b^N/A: not applicable.

### Limitations

This study has limitations. First, it is worth exploring methods to effectively integrate clinical resources with general English resources in transformer-based models. In this study, we explored an approach by pretraining transformer-based models with a clinical corpus (ie, MIMIC-III corpus). However, our results showed that this approach was not efficient. Therefore, new strategies to better integrate medical resources are needed. Second, our clinical STS systems performed better for sentence pairs with high similarity scores (ie, similarity score≥3 in gold standard) whereas, for the sentence pairs with low similarity scores (ie, similarity score<2 in gold standard), our systems still need to be improved. How to address this issue is one of our future focuses.

### Conclusions

In this study, we demonstrated transformer-based models for measuring clinical STS and developed a system that can use various transformer algorithms. Our experiment results show that the RoBERTa model achieved the best performance compared to other transformer models. Our study demonstrated the efficiency of transformer-based models for assessing the semantic similarity for clinical text. Our models and system could be applied to various downstream clinical NLP applications. The source code, system, and pretrained models can be accessed on GitHub [48].

## Appendix

Multimedia Appendix 1 The 1-tailed parametric test results based on Fisher Z-transformation.

## Abbreviations

BERT: Bidirectional Encoder Representations from Transformers
MIMIC-III: Medical Information Mart for Intensive Care
MLM: masked language modeling
n2c2: National Natural Language Processing Clinical Challenges
NIH: National Institutes of Health
NLP: natural language processing
OHNLP: Open Health Natural Language Processing
RoBERTa: Robustly optimized BERT approach
SemEval: semantic evaluation
STS: semantic textual similarity
